# Efficacy of Intraoperative Hypertonic Glucose Solution Administration on Persistent Air Leak After Extended Pleurectomy/Decortication for Malignant Pleural Mesothelioma: A Retrospective Case–Control Study

**DOI:** 10.3389/fonc.2021.767791

**Published:** 2021-12-01

**Authors:** Alberto Testori, Gianluca Perroni, Marco Alloisio, Emanuele Voulaz, Veronica Maria Giudici, Umberto Cariboni, Edoardo Bottoni

**Affiliations:** ^1^ Istituto di Ricovero e Cura a Carattere Scientifico (IRCCS) Humanitas Research Hospital, Division of Thoracic Surgery, Milan, Italy; ^2^ Division of Thoracic Surgery, IRCCS San Raffaele Scientific Institute, Milano, Italy; ^3^ Department of Thoracic Surgery, Tor Vergata University, Rome, Italy; ^4^ Department of Biomedical Sciences, Humanitas University, Milan, Italy

**Keywords:** malignant pleural mesothelioma (MPM), persistent air leak (PAL), prolonged air leak (PAL), hypertonic glucose solution, thoracic surgery (TS)

## Abstract

**Background:**

Persistent air leak is a common complication occurring from 6% to 23% of cases after extended pleurectomy/decortication for malignant pleural mesothelioma. Treatment options for this complication after major lung resection are well documented in literature; nevertheless, lines of evidence in extended pleurectomy/decortication for malignant pleural mesothelioma are absent. The aim of the study is to evaluate the efficacy of intraoperative administration of 50% hypertonic glucose solution in reducing duration of air leak following extended pleurectomy/decortication for malignant pleural mesothelioma.

**Materials and Methods:**

In this retrospective case–control study, we analyzed our electronic health record and selected those patients with a histological diagnosis of malignant pleural mesothelioma who underwent extended pleurectomy/decortication in the period 2013–2021. From 2018, we introduced a lavage with 500 ml of glucose solution at 50% concentration into the chest cavity at the end of the surgical procedure. Patients operated before 2018 were used as the control group. Postoperative glycemia was measured, and patients were followed after hospital discharge until the air leak resolved and the chest tube was removed. Statistical analysis was performed using R software.

**Results:**

A total of 71 patients met our criteria. Treatment and control groups were similar for age, sex, smoking status, number of comorbidities, tumor histotype, and side of disease. Use of hypertonic glucose solution resulted in shorter chest tube maintenance after hospital discharge (*p* = 0.0028). A statistically significant difference (*p* = 0.02) was also found in postoperative glycemia between the treatment (103 g/dl ± 8.9) and control group (98.8 g/dl ± 8.6). Days of hospitalization and chest tube maintenance during hospitalization did not significantly differ between the groups.

**Interpretation:**

Intraoperative administration of 50% hypertonic glucose solution reduced the duration of air leak after hospital discharge. An increase in postoperative glycemia was found in the treatment group, but with no clinical effect. Hypertonic glucose solution is an effective and safe method to manage persistent air leak after extended pleurectomy/decortication for malignant pleural mesothelioma.

## Introduction

Malignant pleural mesothelioma (MPM) is a rare but aggressive tumor arising from pleural surface, with a poor prognosis in most of the cases (1). The main cause of MPM is exposure to asbestos fibers, a silicate mineral largely used in Europe between 1920 and 1970 (2). Use of asbestos fibers is prohibited in most of the countries worldwide; Italy introduced the ban in 1992 (3). Time between inhalation of asbestos fibers and development of MPM can be up to 40 years; therefore, incidence is bound to increase in the next years.

Extra-Pleural Pneumonectomy (EPP) combined with neo-adjuvant chemotherapy and adjuvant radiotherapy represented the standard treatment for carefully selected patients with MPM (4). However, Extended Pleurectomy/Decortication (EPD) has proven to be as effective as EPP in terms of median overall survival and 2-year mortality with lower incidence of death at 30 days (5, 6). Nonetheless, EPD comes with a high rate of postoperative complication, with persistent air leak (PAL) occurring in 6% to 23% of cases (7, 8). As well known, PAL increases hospital stay, intensive care unit readmission, and risk of empyema, and leads to higher in-hospital mortality rate (9).

Data regarding techniques for treatment of PAL comes primarily from lung resective surgery, such as wedge or segmentectomy or lobectomy. Most of the techniques currently available are not applicable to EPD due to the surgical procedure itself: pleural tenting is effective on duration of air leak (2.5 vs 7.2 days; *p* < 0.001) and chest tube maintenance (7.0 vs. 11.2 days; *p* < 0.0001) when performing upper lobectomy and, thus, is unfeasible in EPD since the aim of the procedure is to remove all the pleura (10); transient phrenic nerve paralysis by injecting local anesthetic is useful in reducing pleural space through elevation of diaphragm, but clearly is not possible in EPD since diaphragm is removed and a mesh prosthesis is used in substitution (11); use of intraoperative synthetic sealants is also effective in reducing PAL and chest tube maintenance after lung resection; nonetheless, efficacy in decortication is limited to benign disease (12, 13). As a result, PAL following EPD is usually managed conservatively by applying mild suction to chest tube, then weaning to water seal, and finally using pneumostats for portability if needed (14).

Interestingly, some authors described intraoperative lavage of chest cavity using glucose solution at 50% concentration (GS50) as an effective and inexpensive treatment for reducing PAL and chest tube maintenance. Fujino et al. in 2015 demonstrated safety and efficacy of GS50 in reducing air leak following lung resection and bullectomy for pneumothorax (15). In 2016, Al-Naimi et al. demonstrated a reduction of air leak in 80% of patients at postoperative day (POD) 3 (16). The main limitation of those studies is lack of a control group. In 2008, a prospective randomized controlled trial by Won et al. enrolled 141 patients with primary spontaneous pneumothorax. Patients were divided into three groups based on treatment: thoracoscopy alone, thoracoscopy + glucose solution at 20% concentration, and thoracoscopy + mixture of talc and glucose solution at 20% concentration. The authors found no difference in recurrence rate of pneumothorax and chest tube duration between the three treatment groups (17). Yet, there are two main limitations: concentration of glucose solution was lower in comparison to other studies and criteria for sample size was unclear (β-power unknown).

Until 2018, our department of thoracic surgery routinely used aerosolized fibrin sealant (Tisseel) as the only intraoperative aerostatic agent for EPD. Starting from 2018, we added a lavage of chest cavity with GS50 in EPD for MPM. The aim of this study is to evaluate efficacy of GS50 in reducing duration of air leak and subsequently chest tube maintenance following EPD.

## Materials and Methods

### Selection Criteria

From 2013 to 2021, we retrospectively analyzed our electronical health records and selected those patients with age above 18 years and histological diagnosis of malignant pleural mesothelioma who underwent EPD. Metastatic cancer, severe heart disease, renal impairment, and ASA score > 3 were considered exclusion criteria. Diabetes was not an exclusion criterion. Patients where then divided into two groups: those who did not receive GS50 intraoperatively (before 2018) were used as a control group, whereas those who received GS50 intraoperatively (after 2018) were the treatment group. The study was approved by our hospital ethical committee (MESO1).

### Operative Procedure

After placing a thoracic epidural catheter, a naso-gastric tube (NGT), a central venous catheter (CVC), and an arterial line, the patient was positioned in a lateral fashion. An extended posterolateral thoracotomy with section of latissimus dorsi was performed, and one rib was divided when needed.

After entering the extrapleural plane, the parietal pleura was bluntly separated from the chest wall until a satisfactory mobilization of the lung was reached. Moving towards the mediastinum, the pericardium was removed if macroscopic evidence of invasion was present. Moving downwards, diaphragm was always detached from its insertion. Subsequently, pleura was peeled off from lung starting from the apex to the base. To make the peeling easier, an anesthesiologist was asked to maintain the lung partially inflated.

Once the base was reached, pleura and diaphragm appeared strongly adherent to parenchyma and often the use of a stapler was required to divide and remove the specimen.

After proper hemostasis with gauze compression, the reconstructive phase was initiated by applying a bovine pericardial patch onto the pericardial defect. The patch was fixed using separated stitches, thus preventing pericardial herniation. Suture was not applied in the upper part of the patch in order to avoid cardiac tamponade and adhesion on ascending aorta. A Proceed ® mesh prosthesis was then fixed one or two ribs above the natural insertion of diaphragm, reducing pleural space and allowing the lung to occupy the residual cavity easier.

Once reconstruction was completed, 500 ml of 50% glucose solution was instilled into the chest cavity and aspirated after 2 min of application. Argon beam was then used to improve hemostasis of chest wall. A “cotton-candy like smell” is produced due to high temperature applied on residual glucose. Afterwards, aerosolized Tisseel was applied on lung parenchyma. A satisfactory value of less than 30% in Tidal Volume loss was reached before chest closure. Two chest tubes were positioned into the cavity and connected to a water-sealed chamber with −15 cmH_2_O suction applied on it.

### Postoperative and Outpatient Care

After monitoring in a post-anesthesia care unit, NGT and arterial line were removed if parameters were stable. The patient was then sent to the ward on Post-Operative Day (POD) 0.

Blood test and a chest x-ray (CXR) were routinely performed every day until POD3, then every 2 days if clinical conditions were stable. Sugar blood level was tested only on POD1 and repeated on the following days only if greater than 125 mg/dl. Chest tube suction was applied continuously until POD2 and removed when a satisfactory lung expansion was confirmed at CXR. Due to the high risk of urinary retention, urinary catheter was maintained until thoracic epidural catheter was removed and satisfactory diuresis (>40 ml/h) was reached. Air leak was evaluated according to the five-grade scale by Sang et al. (18). A positive pressure of 10 cmH_2_O was then applied by raising water level in the drainage chamber to test the stability of lung expansion. If no to minimal air leak (grade 0 to 2) and stability of lung expansion at CXR were present after 1 day of positive pressure, discharge was possible. Chest tube was removed if no air leak was evidenced. If minimal air leak persisted, the patient was discharged with a single chest tube and trained to return at our hospital when no air leak was seen coming from Heimlich valve. Confirmation of air leak resolution was confirmed by connecting chest tube to a water-sealed chamber for 30 min, and no suction was applied. If no air leak was noticed, chest tube was removed.

### Statistical analysis

Statistical analysis was performed using R statistical software v 4.0.4. The following data were collected: hospitalization days, chest tube maintenance during hospitalization and after discharge expressed as continuous data (days), glucosate administration expressed as categorical variable (yes or no), age (continuous scale), sex (dichotomic) and postoperative glycemia (continuous scale; expressed in mg/dl), number of comorbidities, smoking habits, side of disease (dichotomic), and pathological stage.

The treatment group was defined as those patients who obtained glucosate administration during surgery; otherwise, they were categorized as the control group. The association of each continuous variable with the categorical ones was evaluated by resorting to the nonparametric Kruskal–Wallis test; comparison of two categorical variables was performed applying a chi-squared test from stats R package.

## Results

A total of 71 patients met our inclusion criterion and were included in the study. Fifty (70.4%) were male and 21 (29.6%) were female. A prevalence of gender in favor to male was therefore present. Mean age at surgery was 65 ± 8 years; 51 patients (71.8%) underwent neoadjuvant chemotherapy.

Treatment (42 patients) and control (29 patients) groups did not differ when comparing age, sex distribution, smoking status, number of comorbidities, neoadjuvant treatment, and side of disease (**Table 1**).

**Table 1:** Clinicopathological characteristics of patients divided in control and treatment group.

		Control group (*n* = 29)	Treatment group (*n* = 42)	*p*-value
Sex, *n* (%)	Male	22 (75.9)	28 (66.7)	0.41
	Female	7 (24.1)	14 (33.3)	
Smoking status, *n* (%)	Never	7 (24.1)	20 (47.6)	0.51
	Current	7 (24.1)	7 (16.7)	
	Former	15 (51.7)	15 (35.7)	
Histotype, *n* (%)	Epithelioid	25 (86.2)	38 (90.5)	0.58
	Biphasic	3 (10.3)	3 (7.1)	
	Sarcomatoid	1 (3.5)	1 (2.4)	
Comorbidities, *n* (%)	0	15 (51.7)	22 (52.4)	0.9
	1	8 (27.6)	13 (30)	
	2	6 (20.7)	7 (16.7)	
Side, *n* (%)	Left	11 (37.9)	22 (52.4)	0.23
	Right	18 (62.1)	20 (47.6)	
Age, mean (SD), years		65 (9)	65 (7)	0.76

**Figure 1:**
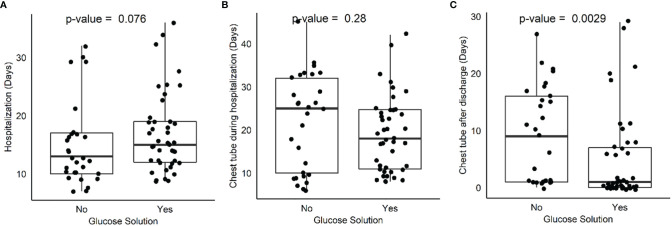
Box plot comparing hospitalization days **(A)**, chest tube maintenance during hospitalization **(B)** and after discharge **(C)** between treatment and control group.

Comparing treatment (42 patients) and control (29 patients) groups, no differences between groups were found regarding days of hospitalization, days of chest tube maintenance during hospitalization, and pathological stage. Interestingly, a difference was found when comparing days of chest tube maintenance after hospital discharge between treatment (15 patients) and control (16 patients) groups (**Table 2**, **Figure 1**). In depth, chest tube was removed earlier in the treatment group (4.95 ± 7.79 versus 9.14 ± 8.4; *p* = 0.0028), thus meaning an earlier resolution of air leak after discharge. Postoperative glycemia was higher in the treatment group (42 patients;103 g/dl ± 8.9; *p* = 0.02) when compared to control (29 patients), but none of the patients had clinically significant symptoms and therefore pharmacological treatment was not necessary.

**Table 2:** Postoperative and pathological characteristics of patients.

		Control group (*n* = 29)	Treatment group (*n* = 42)	*p*-value
Postoperative glycemia, mean (SD), days		98.8 (8.6)	103 (8.9)	0.02
Hospitalization days, mean (SD), days		14.8 (7.1)	17 (6.9)	0.07
Chest tube during hospitalization, mean (SD), days		13.1 (6.9)	14.3 (5.6)	0.13
Chest tube after discharge, mean (SD), days		9.14 (8.4)	4.95 (7.79)	0.0028
pStage, *n* (%)	0	2 (6.9)	2 (4.7)	0.44
	IA	0 (0)	1 (2.4)	
	IB	15 (51.7)	21 (50)	
	IIIA	0 (0)	7 (16.7)	
	IIIB	12 (41.4)	11 (26.2)	

## Discussion

PAL represents a common complication following EPD; nowadays, no evidence is available regarding treatment to reduce air leak duration. Based on our results, intraoperative instillation of GS50 is an effective method to reduce duration of air leak, leading to earlier removal of chest tube. This efficacy was evident after patients were discharged from the hospital, thus potentially reducing risk of infections and improving the quality of life perceived by patients.

Aerosolized Tisseel was used in both treatment and control groups; based on our result, we can say that there is an effect of hypertonic glucose solution on air leak reduction that is visible in the long term. However, the interaction between Tisseel and glucose solution should be explored in experimental models to better understand the effects of these compounds when separated and in combination.

The treatment group had a higher mean glycemic value in comparison to the control group; however, values remained below the threshold of 125 mg/dl. Glucose is therefore minimally absorbed when administered inside the chest cavity, despite what is observed in a previous study published by Tsuboshima et al. (19). A reduction of either quantity or concentration of hypertonic glucose solution administered may limit this phenomenon, and further studies are needed to find the right minimal effective dose.

The mechanism of action leading to reduction of air leak is still unknown. A possible explanation can be found in the inflammation generated by exposure to hypertonic glucose solution and subsequent generation of fibrous adhesion between lung and chest wall that lead to resolution of air leak. In experimental models, cell exposure to high level of glucose leads to overexpression of transforming growth factor beta (TGF-β) and specific surface receptor type I (TβRI) and type II (TβRII) (20) that are the main actors of fibrotic tissue deposition. TGF-β plays a key role in the genesis of adhesion through binding and activating TβRI and TβRII. Activation of receptors leads to phosphorylation of small mother against decapentaplegic (SMAD) intracellular proteins that translocate into the nucleus and act as transcription factors for the deposition of extracellular matrix (ECM). An unbalanced expression of TGF-β isoforms results in the production of altered ECM and subsequent tissue fibrosis (21). In rabbit models the TGF-ß_2_ isoform was effective in generating pleural adhesion, resulting in superior to talc pleurodesis (22, 23). Glucose may therefore be able to determine an altered production of TGF-β isoforms, and this hypothesis can be tested to better understand the mechanism of action.

Another possible explanation is the role of osmotic cell injury secondary to hypertonic glucose solution exposure, leading to precipitation of fibrin and lastly to adhesion between chest wall and lung parenchyma (15).

To our knowledge, this is the first evidence in literature describing efficacy of 50GS in reducing duration of air leak after extended pleurectomy/decortication for malignant pleural mesothelioma. Chest tube was removed earlier in the treatment group, and this difference was significant after patient discharge (*p* = 0.0028). A statistically significant difference was also found in postoperative glycemia (*p* = 0.02), being higher in the treatment group but still below the threshold of 125 mg/dl and therefore with no clinical effect. The mechanism of action leading to reduction of air leak after administering GS50 is still unknown. Due to the efficacy and inexpensiveness, we suggest the use of GS50 for the management of persistent air leak following EPD.

The two main limitations of this study are the retrospective nature of data and the small sample size of patients available. The former does not allow to control potential confounding factors and to evaluate other parameters that were not routinely included in the clinical practice of our ward. The latter prevented us from observing minor differences that may exist between treatment and control groups. A future study with a prospective design and a larger sample size has the potential to validate these results, while the creation of an experimental model may explain the mechanism of action that lies behind the use of hypertonic glucose solution for the treatment of air leak.

## Data Availability Statement

The raw data supporting the conclusions of this article will be made available by the authors, without undue reservation.

## Ethics Statement

The studies involving human participants were reviewed and approved by the Humanitas Research Hospital ethical committee, study code MESO1. The patients/participants provided their written informed consent to participate in this study.

## Author Contributions

AT, GP, UC and EB contributed to the conception and design of the study. MA, VMG, EV, and UC organized the database. VMG and GP contributed to data collection. GP wrote the draft of the manuscript. All authors contributed to manuscript revision, read, and approved the submitted version.

## Conflict of Interest

The authors declare that the research was conducted in the absence of any commercial or financial relationships that could be construed as a potential conflict of interest.

## Publisher’s Note

All claims expressed in this article are solely those of the authors and do not necessarily represent those of their affiliated organizations, or those of the publisher, the editors and the reviewers. Any product that may be evaluated in this article, or claim that may be made by its manufacturer, is not guaranteed or endorsed by the publisher.
